# Supplementary material for: Assessing the applicability of the new Global Lung Function Initiative reference values for the diffusing capacity of the lung for carbon monoxide in a large population set
Source: PLoS One. 2021 Jan 14;16(1):e0245434. doi: 10.1371/journal.pone.0245434 (PMC7808798; doi:10.1371/journal.pone.0245434)
Supplement: S1 Fig — DLCO: Diffusing capacity of the lung for carbon monoxide; ECSC: European Community for Steel and Coal 1993 reference values [2,4]; GLI: Global Lung function Initiative 2017 reference values [6]. (PDF) [file pone.0245434.s001.pdf]

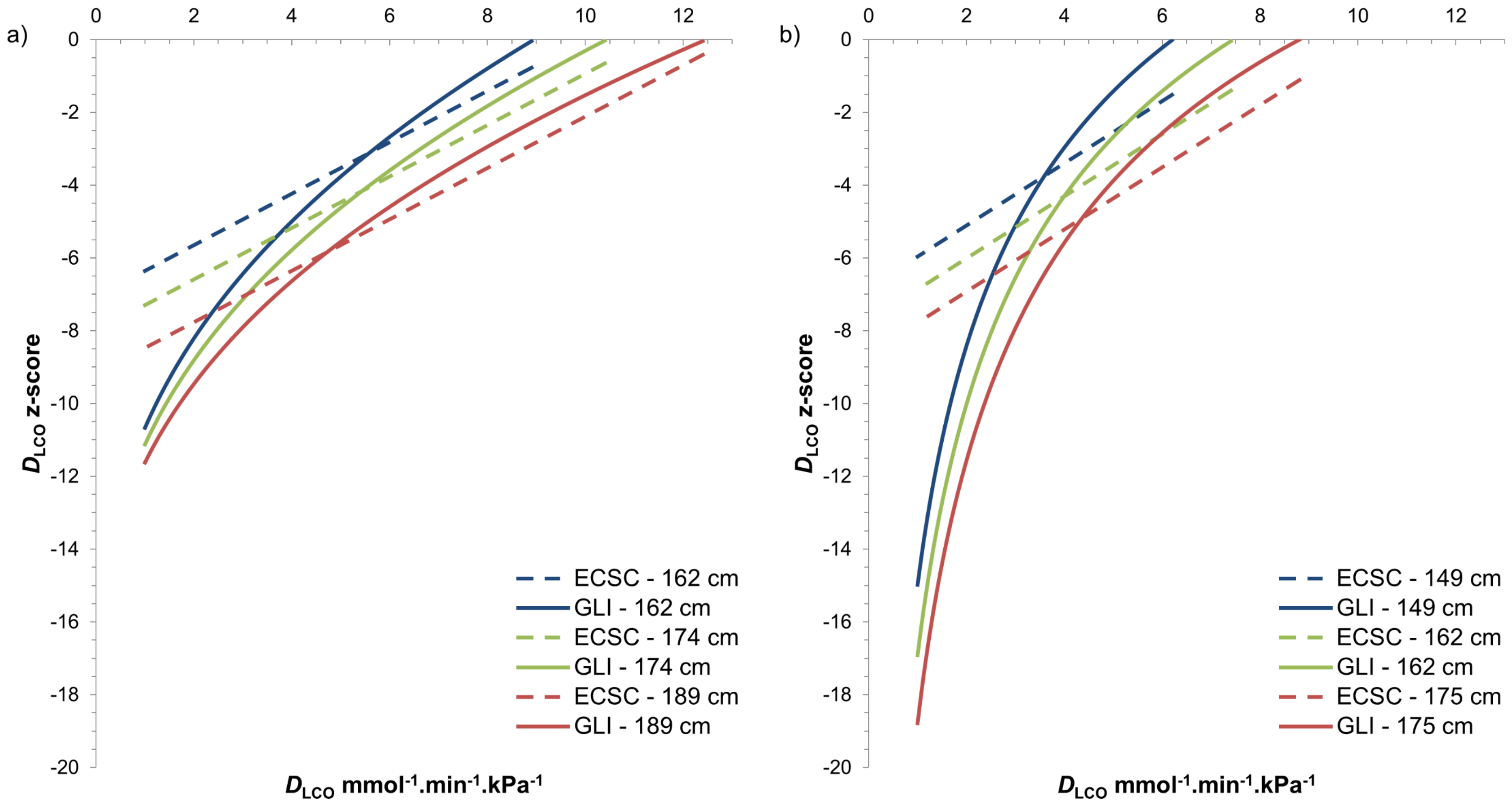

S1 Figure. Mathematical modelling of theorical D_{LCO} as a function of ECSC-93 and GLI-2017 z-scores and height in a 30 years old a) man and b) woman. D_{LCO}: diffusing capacity of the lung for carbon monoxide; ECSC: European Community for Steel and Coal 1993 reference values[1,2]; GLI: Global Lung function Initiative 2017 reference values[3].

REFERENCES

1. Quanjer PH, Dalhuijsen A, Van Zoramen B. Standardized lung function testing. Report working party. Bull Eur Physiopathol Respir. 1983;19 Suppl 5: 1–95.

2. Cotes JE, Chinn DJ, Quanjer PH, Roca J, Yernault J-C. Standardization of the measurement of transfer factor (diffusing capacity). European Respiratory Journal. 1993;6: 41–52. doi:10.1183/09041950.041s1693

3. Stanojevic S, Graham BL, Cooper BG, Thompson BR, Carter KW, Francis RW, et al. Official ERS technical standards: Global Lung Function Initiative reference values for the carbon monoxide transfer factor for Caucasians. European Respiratory Journal. 2017;50: 1700010. doi:10.1183/13993003.00010-2017
