# Supplementary material for: Assessing the applicability of the new Global Lung Function Initiative reference values for the diffusing capacity of the lung for carbon monoxide in a large population set
Source: PLoS One. 2021 Jan 14;16(1):e0245434. doi: 10.1371/journal.pone.0245434 (PMC7808798; doi:10.1371/journal.pone.0245434)
Supplement: S1 Appendix — Contains: S1 Table. Sample sizes, number of classification errors in the random sample and estimated classification error rate on the population of the study according to the ISO 2859–1 standard [24]. ILD: interstitial lung disease. Data are presented as n or %. §Classification error rate estimated for the overall population of the study in each disease group according to the ISO 2859–1 standard [24]. (PDF) [file pone.0245434.s002.pdf]

S1 Appendix. Quality control of the disease group classification

In order to control the quality of the disease-related data collection and the quality of the disease groups classification, we used the ISO 2859-1 standard[1]. We verified the accordance between the diagnosis found in the patient’s medical file and the disease group in which the patient was classified. This was performed on a randomized sample of subjects in each disease groups. Sample sizes were calculated according to the ISO 2859-1 standard with a single sampling plan for normal inspection and an inspection level of II (which is the most frequently used). For each disease group, a classification error rate was estimated, corresponding to the acceptable quality level (AQL) in the ISO 2859-1 standard. The AQL is the acceptable number of non-conformities per 100 units and is determined by the random sample size and the number of errors found in this random sample.

The estimated classification error rate was low (less than or equal to 1% for all disease groups, except for the asthma group where the estimated error rate was equal to 4% (S1 Table in S1 Appendix)).

S1 Table. Sample sizes, number of classification errors in the random sample and estimated classification error rate on the population of the study according to the ISO 2859-1 standard[1].

	Asthma	Chronic bronchitis	Cystic fibrosis	ILD
Subjects, n	527	732	145	2626
Sample size, n	80	80	20	125
Number of errors	6	2	0	2
Estimated classification error rate§	4%	1%	0.65%	0.65%

ILD: interstitial lung disease. Data are presented as n or %. §Classification error rate estimated for the overall population of the study in each disease group according to the ISO 2859-1 standard[1].

References

1. International Organization for Standardization. ISO 2859-1 - Sampling procedures for inspection by attributes -- Part 1: Sampling plans indexed by acceptable quality level (AQL) for lot-by-lot inspection. 1989.
