# Supplementary material for: Assessing the applicability of the new Global Lung Function Initiative reference values for the diffusing capacity of the lung for carbon monoxide in a large population set
Source: PLoS One. 2021 Jan 14;16(1):e0245434. doi: 10.1371/journal.pone.0245434 (PMC7808798; doi:10.1371/journal.pone.0245434)
Supplement: Attachment — Submitted filename: Response to Reviewers.docx [file pone.0245434.s003.docx]

Dear Editor,

Please find below the responses to each point raised during the reviewing process of our manuscript entitled “**Assessing the applicability of the new Global Lung Function Initiative reference values for the diffusing capacity of the lung for carbon monoxide in a large population set**” (authors: P.-M. WARDYN, V. de BROUCKER, C. CHENIVESSE, A. SOBASZEK, R. VAN BULCK, T. PEREZ, J.-L. EDME, S. HULO):

**EDITOR COMMENTS:**

**I would recommend the change of the title of the manuscript to: „Assessing the applicability of the new Global Lung Function Initiative reference values for the diffusing capacity of the lung for carbon monoxide in a large population set“**

The title was changed from “Application of the new Global Lung Function Initiative reference values for the diffusing capacity of the lung for carbon monoxide to a large population” to „Assessing the applicability of the new Global Lung Function Initiative reference values for the diffusing capacity of the lung for carbon monoxide in a large population set“

**Please describe in detail how the used tests were evaluated for inclusion/exclusion into the study.**

In order to describe in detail how the used tests were evaluated for inclusion/exclusion into the study, the following paragraph was added in “materiel and methods”, in the “study population” part:

“For each test, PFTs indices values and patient’s characteristics (sex, age, height, weight) are recorded in the laboratory database and a test report including patient’s medical history is created using Microsoft Access® which is also recorded in the laboratory database. For the tests’ inclusion, data from the PFT performed between November 15th 2012 and May 30th 2016, and including the indices mentioned above, were extracted to a Microsoft Excel® spreadsheet by the laboratory engineer. Then tests meeting exclusion criteria were excluded using the statistical software SAS® (version 9.4; Statistical Analysis System).”

**Add actual p-values to Table 1. You mentioned that “GLI-2012 produce significantly lower median z-scores for FEV1, FVC and FEV1/FVC 180 than ECSC, all groups combined, in both genders (table 2).” but no p-values were cited. Please include actual p-values in the Results. Also for Table 3 include actual p-values and refer them in the text where you state significant differences. Please do the same for Table 4.**

Actual p-values were added to Table 1, 3 and 4. Actual p-values were added in the Results. We set the limit to p < 0.0001.

**JOURNAL REQUIREMENTS:**

**1. Please ensure that your manuscript meets PLOS ONE's style requirements, including those for file naming. The PLOS ONE style templates can be found at**

[**https://journals.plos.org/plosone/s/file?id=wjVg/PLOSOne_formatting_sample_main_body.pdf**](https://journals.plos.org/plosone/s/file?id=wjVg/PLOSOne_formatting_sample_main_body.pdf) **and**

[**https://journals.plos.org/plosone/s/file?id=ba62/PLOSOne_formatting_sample_title_authors_affiliations.pdf**](https://journals.plos.org/plosone/s/file?id=ba62/PLOSOne_formatting_sample_title_authors_affiliations.pdf)

 We ensured that our manuscript meets PLOS ONE's style requirements. Affiliation components’ order and the reference to S1 appendix and table in “material and method“ were corrected.

**2. We note that you have indicated that data from this study are available upon request. PLOS only allows data to be available upon request if there are legal or ethical restrictions on sharing data publicly. For information on unacceptable data access restrictions, please see** [**http://journals.plos.org/plosone/s/data-availability#loc-unacceptable-data-access-restrictions**](http://journals.plos.org/plosone/s/data-availability#loc-unacceptable-data-access-restrictions)**.**

**In your revised cover letter, please address the following prompts:**

**a) If there are ethical or legal restrictions on sharing a** **de-identified data set, please explain them in detail (e.g., data contain potentially identifying or sensitive patient information) and who has imposed them (e.g., an ethics committee). Please also provide contact information for a data access committee, ethics committee, or other institutional body to which data requests may be sent.**

**b) If there are no restrictions, please upload the minimal anonymized data set necessary to replicate your study findings as either Supporting Information files or to a stable, public repository and provide us with the relevant URLs, DOIs, or accession numbers. Please see** [**http://www.bmj.com/content/340/bmj.c181.long**](http://www.bmj.com/content/340/bmj.c181.long) **for guidelines on how to de-identify and prepare clinical data for publication. For a list of acceptable repositories, please see** [**http://journals.plos.org/plosone/s/data-availability#loc-recommended-repositories**](http://journals.plos.org/plosone/s/data-availability#loc-recommended-repositories)**.**

**We will update your Data Availability statement on your behalf to reflect the information you provide.**

We explained why we cannot share the de-identified data set in the cover letter in the following paragraph:

“Please note that that we cannot share the de-identified data set for legal reasons on the Research Ethics Board of the University Hospital of Lille demand. The data set includes sensitive medical information about included patients as medical history and PFT results. Sex, age, height and weight are also included in the data set, which can potentially allow the patients’ identification. Data requests may be sent to the Research Ethics Board of the University Hospital of Lille (contact: +33 3.20.44.41.65 / [cppnordouestiv@univ-lille2.fr)](mailto:cppnordouestiv@univ-lille2.fr)).”

**3. PLOS requires an ORCID iD for the corresponding author in Editorial Manager on papers submitted after December 6th, 2016. Please ensure that you have an ORCID iD and that it is validated in Editorial Manager. To do this, go to ‘Update my Information’ (in the upper left-hand corner of the main menu), and click on the Fetch/Validate link next to the ORCID field. This will take you to the ORCID site and allow you to create a new iD or authenticate a pre-existing iD in Editorial Manager. Please see the following video for instructions on linking an ORCID iD to your Editorial Manager account:** [**https://www.youtube.com/watch?v=_xcclfuvtxQ**](https://www.youtube.com/watch?v=_xcclfuvtxQ)

 The ORCID iD was validated in Editorial Manager.

**4. We note that you have included the phrase “data not shown” in your manuscript. Unfortunately, this does not meet our data sharing requirements. PLOS does not permit references to inaccessible data. We require that authors provide all relevant data within the paper, Supporting Information files, or in an acceptable, public repository. Please add a citation to support this phrase or upload the data that corresponds with these findings to a stable repository (such as Figshare or Dryad) and provide and URLs, DOIs, or accession numbers that may be used to access these data. Or, if the data are not a core part of the research being presented in your study, we ask that you remove the phrase that refers to these data.**

We added to the **supporting Information files** the S1 figure entitled “Mathematical modelling of theorical *D*_LCO_ as a function of ECSC-93 and GLI-2017 z-scores and height in a 30 years old a) man and b) woman” and replace the “data not shown” seen in “Discussion” with a reference to this S1 figure as follow: “Mathematical modelling showed an inversion of the GLI/ECSC z-scores ratio when *D*_LCO_ is extremely decreased (see S1 figure)”.

Hoping this revised version of our work will meet with your approval,

Sincerely yours,

Pierre-Marie WARDYN, MD, Virginie de BROUCKER, MD, Cécile CHENIVESSE, MD, PhD, Annie SOBASZEK, MD, PhD, Richard VAN BULCK, Thierry PEREZ, MD, Jean-Louis EDME, PhD, Sébastien HULO, MD, PhD.
