# Supplementary material for: Assessing the applicability of the new Global Lung Function Initiative reference values for the diffusing capacity of the lung for carbon monoxide in a large population set
Source: PLoS One. 2021 Jan 14;16(1):e0245434. doi: 10.1371/journal.pone.0245434 (PMC7808798; doi:10.1371/journal.pone.0245434)
Supplement: Attachment — Submitted filename: Response to Reviewers def.docx [file pone.0245434.s004.docx]

Dear Editor,

Please find below the responses to each point raised during the reviewing process of our manuscript entitled “**Assessing the applicability of the new Global Lung Function Initiative reference values for the diffusing capacity of the lung for carbon monoxide in a large population set**” (authors: P.-M. WARDYN, V. de BROUCKER, C. CHENIVESSE, A. SOBASZEK, R. VAN BULCK, T. PEREZ, J.-L. EDME, S. HULO):

**EDITOR COMMENTS:**

**Please do the suggested minor revision by the reviewer or write a detailed rebuttal prior to publication.**

Please find below our responses to the suggested minor revision by the reviewer.

**REVIEWERS' COMMENTS:**

**2. Is the manuscript technically sound, and do the data support the conclusions?**

**The manuscript must describe a technically sound piece of scientific research with data that supports the conclusions. Experiments must have been conducted rigorously, with appropriate controls, replication, and sample sizes. The conclusions must be drawn appropriately based on the data presented.**

**Reviewer #1: Partly**

In our study, we aimed not to verify the interest of measuring *D*_LCO_ for specific pulmonary diseases but to assess the applicability of the GLI-2017 reference values for *D*_LCO_ of adult subjects by comparing the GLI-2017 reference values to the ECSC-93 equations currently used in a large population set. The data in this study support our conclusions: the GLI-2017 reference values are more suitable to our population than the ECSC-93 equations and differences between the 2 equations sets depend on the severity of *D*_LCO_ impairment in the Interstitial Lung Disease group.

**For my point of view there is inconsistency in the definition of the primary disease. Was it "patients with symptoms compatible with one of the diseases" or clinical diagnosis (confirmed or highly suspected diagnoses, established by the referring physician) or "diagnosis were checked from patient files in random samples of each groups"? That should be better characterised and if the disease was not properly validated the groups should be named accordingly.**

The diagnoses were confirmed or highly suspected, established by the referring physician (pulmonologist or internist) prescribing the PFT, and to ensure data quality, we checked the diagnoses from patient files in random samples of each groups.

This point was clarified in materials and methods (“In order to ensure data quality, diagnoses were checked from patient files in random samples of each groups”) and discussion (“This retrospective study uses confirmed or highly suspected diagnoses, established by the referring physician (pulmonologist or internist) prescribing the PFT, and recorded in the laboratory database. However, in order to ensure data quality, diagnoses were checked from patient files in random samples of each groups.”).

**The 15% of normal lung function in the COPD group and 7% restriction without obstruction should be explained in the methods and addressed in the discussion.**

The fact that there are 15% of normal lung function in the COPD group and 7% restriction without obstruction have been explained in materials and methods (“Selected PFTs could have been performed at any stage of the concerned disease, from initial evaluation to advanced stage”) and addressed in the discussion (“We included tests performed at every stage of the diseases, which can be very early, explaining why some PFTs from patients with COPD are classified as normal (16.5% in women and 15% in men). On the other hand, some are classified as restrictive without obstruction (3.1% in women and 6.8% in men with COPD). This can append in case of an early stage COPD evaluation in a patient with a concomitant disease which has not been clinically detected by the prescribing physician.”)

It is also worth noticing that we aimed not to verify the interest of measuring *D*_LCO_ for specific pulmonary diseases but to assess the applicability of the GLI-2017 reference values for *D*_LCO_ of adult subjects.

Hoping this revised version of our work will meet with your approval,

Sincerely yours,

Pierre-Marie WARDYN, MD, Virginie de BROUCKER, MD, Cécile CHENIVESSE, MD, PhD, Annie SOBASZEK, MD, PhD, Richard VAN BULCK, Thierry PEREZ, MD, Jean-Louis EDME, PhD, Sébastien HULO, MD, PhD.
