# Supplementary material for: Assessing the applicability of the new Global Lung Function Initiative reference values for the diffusing capacity of the lung for carbon monoxide in a large population set
Source: PLoS One. 2021 Jan 14;16(1):e0245434. doi: 10.1371/journal.pone.0245434 (PMC7808798; doi:10.1371/journal.pone.0245434)
Supplement: Attachment — Submitted filename: Rew II.docx [file pone.0245434.s005.docx]

**Your explanation**: “The fact that there are 15% of normal lung function in the COPD group and 7%

restriction without obstruction have been explained in materials and methods

(“Selected PFTs could have been performed at any stage of the concerned disease,

from initial evaluation to advanced stage”) and addressed in the discussion (“We

included tests performed at every stage of the diseases, which can be very early,

explaining why some PFTs from patients with COPD are classified as normal (16.5% in

women and 15% in men). On the other hand, some are classified as restrictive without

obstruction (3.1% in women and 6.8% in men with COPD).”

GOLD 2021: The FEV1/FVC<0,70 is required for the diagnosis of CPOD, normal lung function or restriction without obstruction are ruling out COPD as diagnosis. Your explanation is not in accordance with actual guidelines. It does not matter whether it is important to the whole article, but this group can not be declared as COPD patients.

**Study population**

We selected data of a control group and patients with symptoms compatible with one of four diseases* (asthma, chronic obstructive pulmonary disease (COPD), cystic fibrosis, and interstitial lung diseases (ILD))

*not with the diagnosed disease!

**Groups creation**

Selected PFTs came from 4 groups of patients with symptoms compatible* with one of the following diseases: asthma, COPD, cystic fibrosis, and ILD.

*not with the diagnosed disease!

**Discussion**

We have included PFTs from patients with one defined disease* (asthma, COPD, cystic fibrosis or ILD).

*now we have a defined disease?

This retrospective study uses confirmed or highly suspected diagnoses*, established by the referring physician (pulmonologist or internist) prescribing the PFT.

*Here we have confirmed or highly suspected disease!

We included tests performed at every stage of the diseases, which can be very early, explaining why some PFTs from patients with COPD are classified as normal* (16.5% in women and 15% in men).

*There is no early COPD with normal lung function or add a reference

On the other hand, some are classified as restrictive without obstruction (3.1% in women and 6.8% in men with COPD). This can append* in case of an early-stage COPD** evaluation in a patient with a concomitant disease which has not been clinically detected by the prescribing physician.

*append, maybe happened?

**please correct or reference

Dear colleagues,

I value your work and believe that it is worth of publishing but not with the wrong definition of the COPD. I would suggest you correct the problems I have note with the asterisks. I see two solutions: to rename the COPD group or to exclude the patients without obstruction from the statistical analyze.

Hope that
