# Supplementary material for: Assessing the applicability of the new Global Lung Function Initiative reference values for the diffusing capacity of the lung for carbon monoxide in a large population set
Source: PLoS One. 2021 Jan 14;16(1):e0245434. doi: 10.1371/journal.pone.0245434 (PMC7808798; doi:10.1371/journal.pone.0245434)
Supplement: Attachment — Submitted filename: Response to Reviewers.docx [file pone.0245434.s006.docx]

Dear Editor,

Please find below the responses to each point raised during the reviewing process of our manuscript entitled “**Assessing the applicability of the new Global Lung Function Initiative reference values for the diffusing capacity of the lung for carbon monoxide in a large population set**” (authors: P.-M. WARDYN, V. de BROUCKER, C. CHENIVESSE, A. SOBASZEK, R. VAN BULCK, T. PEREZ, J.-L. EDME, S. HULO):

**EDITOR COMMENTS:**

**Please resolve the issue raised by the reviewer.**

Please find below the solution we proposed in order to resolve the issue raised by the reviewer.

**REVIEWERS' COMMENTS:**

We would like to thank the reviewer for his interest in our work.

**This is the explanation of my decision and what I believe should be corrected:**

**Your explanation: “The fact that there are 15% of normal lung function in the COPD group and 7% restriction without obstruction have been explained in materials and methods (“Selected PFTs could have been performed at any stage of the concerned disease, from initial evaluation to advanced stage”) and addressed in the discussion (“We included tests performed at every stage of the diseases, which can be very early, explaining why some PFTs from patients with COPD are classified as normal (16.5% in women and 15% in men). On the other hand, some are classified as restrictive without obstruction (3.1% in women and 6.8% in men with COPD).”**

**GOLD 2021:** **The FEV1/FVC<0,70 is required for the diagnosis of CPOD, normal lung function or restriction without obstruction are ruling out COPD as diagnosis. Your explanation is not in accordance with actual guidelines. It does not matter whether it is important to the whole article, but this group can not be declared as COPD patients.**

In order to clarify our definition of our “COPD group” and to be in accordance with the GOLD 2021, we opted for your second proposition mentioned below: we replaced the name of the "COPD" group with the term "chronic bronchitis". We included the following sentence in the methods in the “group creation” part: “The chronic bronchitis group included patients with symptoms compatible with chronic bronchitis (with a FEV_1_/FVC ratio ≥ 0.7) or with chronic obstructive pulmonary disease (COPD) (with a FEV_1_/FVC ratio < 0.7 according to the GOLD 2021 report[1])”.

**Study population**

**We selected data of a control group and patients with symptoms compatible with one of four diseases* (asthma, chronic obstructive pulmonary disease (COPD), cystic fibrosis, and interstitial lung diseases (ILD))**

***not with the diagnosed disease!**

We harmonized the text with the formulation “patients with symptoms compatible with […]” and replaced COPD by chronic bronchitis, and so, we modified the sentence as follow: “We selected data of a control group and patients with symptoms compatible with with one of four diseases* (asthma, chronic bronchitis, cystic fibrosis, and interstitial lung diseases (ILD))”.

**Groups creation**

**Selected PFTs came from 4 groups of patients with symptoms compatible* with one of the following diseases: asthma, COPD, cystic fibrosis, and ILD.**

***not with the diagnosed disease!**

We harmonized the text with the formulation “patients with symptoms compatible with […]” and replaced COPD by chronic bronchitis, and so, we modified the sentence as follow: “Selected PFTs came from 4 groups of patients with symptoms compatible with one of the following diseases: asthma, chronic bronchitis, cystic fibrosis, and ILD.”

**Discussion**

**We have included PFTs from patients with one defined disease* (asthma, COPD, cystic fibrosis or ILD).**

***now we have a defined disease?**

We modified the formulation by “patients with symptoms compatible with one of four diseases (asthma, chronic bronchitis, cystic fibrosis or ILD)” in order to harmonize the text of the discussion section with the methods section mentioned above.

**This retrospective study uses confirmed or highly suspected diagnoses*, established by the referring physician (pulmonologist or internist) prescribing the PFT.**

***Here we have confirmed or highly suspected disease!**

We modified the formulation by “This retrospective study uses highly suspected diagnoses […]”.

**We included tests performed at every stage of the diseases, which can be very early, explaining why some PFTs from patients with COPD are classified as normal* (16.5% in women and 15% in men).**

***There is no early COPD with normal lung function or add a reference**

The sentence was modified by “Some PFTs from the chronic bronchitis group are classified as normal (16.5% in women and 15% in men). There are 2 main reasons. First, we included tests performed at every stage of the diseases, including patients with mild symptoms of chronic bronchitis without obstruction. Secondly, we used z-score to define obstruction according to the 2005-ATS/ERS recommendations for lung function test interpretation[2] (z-score of FEV_1_/FVC ratio < -1.645 and TLC z-score ≥ -1.645). Using the GOLD 2021 definition for COPD (FEV_1_/FVC < 0.7)[1] could have led us to classify fewer PFTs as normal in the chronic bronchitis group in our sample due to the fact that a FEV_1_/FVC ratio < 0.7 can be associated with a z-score of the FEV_1_/FVC ratio > -1.645”.

**On the other hand, some are classified as restrictive without obstruction (3.1% in women and 6.8% in men with COPD). This can append* in case of an early-stage COPD** evaluation in a patient with a concomitant disease which has not been clinically detected by the prescribing physician.**

***append, maybe happened?**

This typing error was corrected.

****please correct or reference**

The sentence was changed to “On the other hand, some PFTs in the chronic bronchitis group were classified as restrictive without obstruction (3.1% in women and 6.8% in men with chronic bronchitis). This may happen in case of chronic bronchitis evaluation in a patient with a concomitant disease which has not been clinically detected by the prescribing physician”.

**I value your work and believe that it is worth of publishing but not with the wrong definition of the COPD. I would suggest you correct the problems I have note with the asterisks. I see two solutions: to rename the COPD group or to exclude the patients without obstruction from the statistical analyze of COPD group.**

As mentioned above, we renamed the COPD group as the chronic bronchitis group.

Hoping this revised version of our work will meet with your approval,

Sincerely yours,

Pierre-Marie WARDYN, MD, Virginie de BROUCKER, MD, Cécile CHENIVESSE, MD, PhD, Annie SOBASZEK, MD, PhD, Richard VAN BULCK, Thierry PEREZ, MD, Jean-Louis EDME, PhD, Sébastien HULO, MD, PhD.

**REFERENCES**

1.	Global initiative for chronic obstructive lung disease (GOLD). Global strategy for the diagnosis, management, and prevention of chronic obstructive pulmonary disease (2021 report). 2020. Available: https://goldcopd.org/2021-gold-reports/

2.	Pellegrino R, Viegi G, Brusasco V, Crapo RO, Burgos F, Casaburi R, et al. Interpretative strategies for lung function tests. European Respiratory Journal. 2005;26: 948–968. doi:10.1183/09031936.05.00035205
